# Penetration of Fosfomycin into IPEC-J2 Cells in the Presence or Absence of Deoxynivalenol

**DOI:** 10.1371/journal.pone.0075068

**Published:** 2013-09-06

**Authors:** Guadalupe Martínez, Denisa S. Pérez, Alejandro L. Soraci, María O. Tapia

**Affiliations:** 1 Centro de Investigación Veterinaria de Tandil CIVETAN-CONICET-FCV, Tandil, Buenos Aires, Argentina; 2 Laboratorio de Toxicología, Facultad de Ciencias Veterinarias, Universidad Nacional del Centro de la Provincia de Buenos Aires, Tandil, Buenos Aires, Argentina; Northeast Agricultural University, China

## Abstract

Fosfomycin (FOS) is an antibiotic used in pig farms for treatment and prevention of infections caused by resistant bacteria during the post-weaning period. Antibiotics and non-toxic concentrations of mycotoxins, such as deoxynivalenol (DON) are frequently found in the diet of animals. These compounds can establish interactions in the intestinal tract, which could affect and/or modify the penetration of FOS to enterocytes. The aim of this study was to determine the penetration of FOS into IPECJ-2 cells, a cell line derived from the small intestine of piglets, in the presence and absence of DON. The results from this study showed that there was statistically significant difference in the intracellular concentration of FOS between cells incubated with 580 µg/ml FOS and cells incubated with 580 µg/ml FOS and 1 µg/ml DON. The C_max_ of the intracellular antibiotic in the culture plates incubated with FOS in absence of DON was 45.81 µg/ml with a t_max_ of 4 h. When IPEC-2 cells were incubated with FOS and DON the C_max_ was 20.06 µg/ml and the t_max_ was 30 min. It is concluded that the non-toxic concentration of DON on IPEC-J2 cells after short-term exposure, interferes with the pharmacokinetics of the antibiotic.

## Introduction

Fosfomycin (FOS), cis-1,2-epoxyphosphonic acid, is an antibacterial drug discovered in 1961. It has a low molecular weight (138.059 Da) and its chemical structure is similar to that of phosphoenol-pyruvate. However, two symmetrical phosphate radicals, connected to a three carbon structure give this drug a unique chemical structure unrelated to other antibacterial families and no cross-resistance with other antibiotics has been reported [1], [2]. It has a wide spectrum antibacterial activity against gram-positive and gram-negative bacteria [3], [4], [5], [6]. Orally, FOS is used in its calcium salt form. Calcium FOS has poor intestinal absorption, reaching low concentrations in serum [7]. In clinical practice, FOS represents a potential alternative for the treatment of infections caused by resistant bacteria in weaning piglets [8]. On the other hand, animals could be affected by the presence of anti-nutritional compounds in the diet, for example, mycotoxins. Deoxinivalenol (DON) belongs to the trichothecene family of mycotoxins, which are produced by *Fusarium* sp., fungi that commonly contaminate cereal grains, such as wheat and maize [9]. DON is of major importance in animal feeding, mainly because of its frequent occurrence in cereal grains, being porcine the most sensitive species to the toxic effects of this mycotoxin [10], [11]. Roigé et al. carried out a study to identify the mycotoxins and mycobiota present in samples of wheat (as bran) and corn (as dry grain or fermented feed), which are main ingredients of feedstuffs used in cattle and pig farms in the South of Buenos Aires Province, Argentina. *Fusarium* (47%) was the most frequent fungus isolated from corn. DON was detected in 59% of corn samples, in 45% of wheat samples and in 38% of the silage samples [12]. In Europe, DON is the most prevalent trichothecene in crop production and it contaminates common cereal-based diets [13], [14].

Contaminations in the diet with high concentrations of DON induce vomiting, diarrhea, immunosuppression and malabsorption of nutrients [15], [16], [17]. The mechanism of action of DON is based on the inhibition of protein synthesis in the cells. Furthermore, DON affects growth and function of intestinal epithelial cells by several pathological mechanisms, including activation of cellular signaling and ribosomal stress [18].

Following ingestion of DON-contaminated food and antibiotic in the diet, the intestine is exposed to the interaction between both compounds. The result of this interaction could adversely affect the effectiveness of antibiotic by interfering with the penetration of the drug into the enterocytes or by affecting intestinal absorption. The integrity of the intestine allows efficient nutrient absorption, acts as a physical barrier for pathogens and toxins and participates in the innate immune response [19], [20], [21], [22].

IPEC-J2 is a non-transformed columnar epithelial cell line originated from the mid-jejunum of a neonatal piglet [23]. Recently, IPEC-J2 cells were used to demonstrate that high concentrations of DON reduced the viability of porcine intestinal epithelial cells, especially when applied basolaterally [11], [24], [25]. Consequently, IPEC-J2 cells are a good model for studying whether the penetration of FOS into enterocytes may be affected by the presence of low concentrations of DON in the diet. Given the high homology in porcine and human intestinal structure and function, and presumably in enterocytes as well [23], [26], studies performed with IPEC-J2 cells may provide valuable insights on the possible effects of interactions between mycotoxins and antibiotics on human intestinal mucosa.

The aim of this study was to determine the penetration of FOS into IPECJ-2 cells in the presence and absence of DON.

## Materials and Methods

### IPEC-J2 cells

IPEC-J2 cells were kindly provided by Dr. Anthony Blikslager (North Carolina State University, Raleigh, NC) [27]. The cells were cultured in Dulbecco’s modified Eagle medium: nutrient mixture F-12 (Ham) (1:1) with GlutaMAX™-I (DMEM/F12) (Invitrogen ™ Life Technologies, Carlsbad, CA, USA) supplemented with 20% fetal bovine serum, 1% insulin-transferring-selenium supplements (Invitrogen), 1% epidermal growth factor (Invitrogen) and 1% penicillin-streptomycin (penicillin 10,000 U/ml and streptomycin 100 mg/ml; Invitrogen) in a humidified atmosphere of 5% CO_2_ at 37°C. The cells were routinely seeded at a density of 2×10^5^/ml with 5 ml medium in plastic tissue culture flasks (25 cm^2^,Greiner Bio One, Frickenhausen, Germany) and passaged every 4 days for a maximum of 4 times (IPEC-J2 passages 20-24).

IPEC-J2 cells were seeded into 24-well plates at a concentration of 1.2×10^5^/ml in a final volume of 1 ml per well. Cells reached a confluent monolayer within 4 days and were then used in all experiments.

### Cell exposure to antibiotic

IPEC-J2 cells were exposed to 580 µg/ml calcium FOS (98.9% of purity; Bedson Laboratory, Pilar, Buenos Aires, Argentina). This concentration was estimated based on the fact that in weaning piglets, calcium FOS is administered at 30 mg/kg body weight, thus the estimated total dose for a 15 kg-piglet is 450 mg. Eighty percent of the administered drug remains in the gut [8]. Therefore, only 360 mg of FOS could reside in the gut. In addition, because the intestinal volume of a 15 kg-piglet is 622 ml [28], it is estimated that 580 µg of FOS can be found in each milliliter of intestinal volume. In conclusion, it was determined that the appropriate concentration of FOS to use in the assays was 580 ppm (µg/ml).

### Cell exposure to mycotoxin

IPEC-J2 cells were exposed to 1 µg/ml DON with 580 µg/ml calcium FOS. This concentration was determined starting from low concentration of DON (less than 3 ppm DON in the diet) [15]. For this experiment, it was assumed that DON is completely bio-accessible and when ingested in one meal by a 15 kg-piglet that consumes 800 g food/day, DON is diluted in 622 ml of gastrointestinal fluid. Purified DON (D0156; 040M4062 Lot Number; Sigma-Aldrich, Germany) was diluted in absolute methanol grade H.P.L.C. (99.9%, Sintorgan®) to prepare a 1 ppm stock solution. At the time of performing the assays, tubes with 1 ppm DON were evaporated to dryness and diluted with antibiotic in isotonic saline before addition to the culture plates.

### Intracellular penetration of fosfomycin

Three groups of cell cultures were used. Group I was constituted by the IPEC-J2 cultures without antibiotic or mycotoxin (control group). For Group II, cell culture plates were incubated with FOS and for Group III, IPEC-J2 cells were incubated with a combination of FOS and DON. The antibiotic and mycotoxin were diluted in isotonic saline and were added to corresponding cell culture plates at a concentration of 580 ppm and 1 ppm, respectively. Cells in the control group were incubated with 1 ml of isotonic saline. Cells were maintained in an atmosphere of 5% CO_2_ at 37°C for different periods of time (0, 5, 10, 15 and 30 min, 1–4, 6, 8,12 and 24 h).

### Cell viability

Cell concentration and viability were determined by trypan blue exclusion assay in the three groups after 24 h of incubation of the IPEC-J2 cells spiked with FOS and/or DON. The isotonic saline with FOS or FOS plus DON from the culture plates was discarded and tripsin was added to the cells. Once cells were detached, the culture medium containing fetal bovine serum was added to the wells in order to stop the activity of the trypsin. Aliquots of 10 μl of cell suspension and 10 μl of 0.4% tripan blue solution were mixed. The concentration and cell viability, (number of living cells/number of total cells) x 100, were determined in a hemocytometer Neubauer chamber under an optical microscope.

### Extraction of fosfomycin

After incubation, cells were washed twice with 1 ml HPLC water to eliminate the free antibiotic that was not taken up by the cells. Then, 1 ml HPLC water was added to each well of the culture plates. Cells were lysed by sonication for 30 min to release the intracelullar antibiotic to the HPLC water. After centrifugation at 10.000 rpm at 4°C for 6 min, the supernatant was filtered through 0.22 µ nylon filters, placed in vials and analyzed by high performance liquid chromatography-mass-mass (HPLC MS-MS) according to the method described by Soraci et al. [8]. The HPLC-MS/MS system was from Thermo Electron Corporation (San Jose, CA, USA), consisting of a Finnigan Surveyor auto sampler, a Finnigan Surveyor MS quaternary pump. The detector was a Thermo Quantum Discovery Max triple quadrupole mass spectrometer, equipped with electrospray (ESI) ion source. Nitrogen was used as nebulizer and sheath gas was obtained through a nitrogen generator from Peak Scientific Ltd. (Inchinnan, Scotland). Data processing was done using Xcalibur software, also from Thermo. The mass spectrometer was operated in negative ionization mode. The tuning parameters were optimized with 10 µg/ml individual aqueous solutions of fosfomycin directly infused in the ion source by means of a syringe pump at 10 µl/min, with influence of a mobile phase delivered from the LC pump through a T connection to give the corresponding chromatographic flow rate. The spray voltage was set to –3800 eV, the capillary temperature was 350°C and argon 99.999% purity was used for Collision Induced Dissociation (CID) at 1.6 m Torr in the collision cell. Source CID energy was set to –8eV. Fosfomycin detection and quantification were achieved by single reaction monitoring of transitions m/z 137° → 79 with an optimized collision energy of 25. Lower limit of quantification (LOQ) was 0.1 µg/ml, a parameter which was thoroughly studied by our research group in different tissues of piglets, such as muscle, liver, gut and kidney.

The average volume of IPEC-J2 cells was calculated by multiplying the concentration of cells in each well of the culture plates by the average volume of intracellular water in mononuclear cells (3.75×10-^6^µl) [29].

### Statistical analysis

Data were analyzed by Student's t test and *p* values were calculated using SAS. Mean values represent triplicate measurement of at least 3 independent experiments. Significant differences between cells treated with FOS and cells treated with FOS and DON were considered when *p* < 0.05.

## Results

Dead cells were not observed in the groups incubated with isotonic saline (control group), with FOS and with FOS and DON after 24 h of incubation. Differences in the percentage of cell viability were not observed (p>0.05) between the groups. On the other hand, the mean concentration of cells in each well of the culture plates, as determined by trypan blue exclusion assay, was 0.9×10^5^ cells/ml for the three groups.

**Figure 1:**
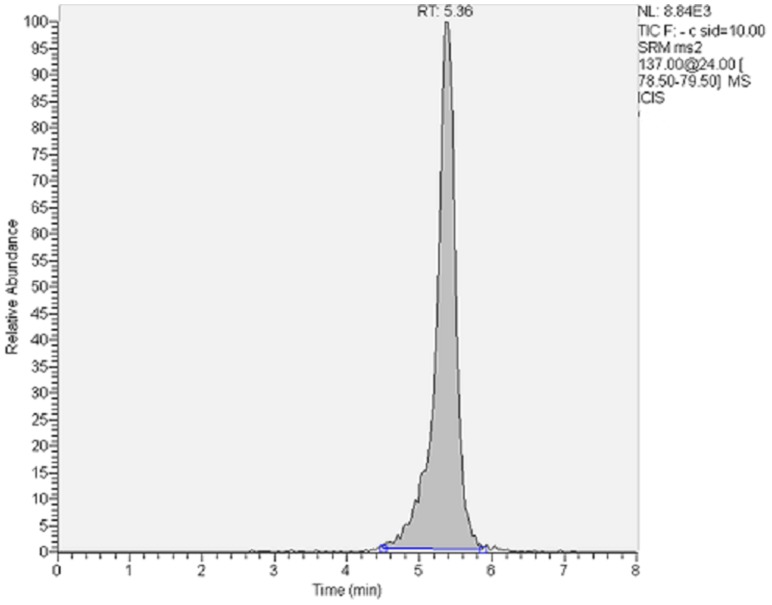
Intracellular concentration of FOS after treatment with 580 ppm FOS and 1 ppm DON in IPEC-J2 cells after 4 h of incubation.

The concentrations of intracellular FOS detected by HPLC MS-MS exceeded thoroughly the limit of quantification for this drug. Furthermore, matrix effects were not observed during the determination of FOS. The chromatogram in figure 1 shows the high resolution and the peak shape obtained for the assays performed with 580 ppm FOS and 1 ppm DON.

The comparison between the antibiotic group vs the antibiotic and mycotoxin group allowed us to determine that DON produces a significant effect in the penetration of FOS into the cells.

The maximum concentration (C_max_) of intracellular antibiotic in the culture plates incubated with 580 µg/ml calcium FOS was 45.81. It was determined that the maximum concentration (t_max_) was reached after four hours of incubation. After this time, the concentration of intracellular antibiotic decreased to 23.48 µg/ml at 24 h of incubation. FOS slowly accumulated within IPEC-J2 cells and only 7.89% of the antibiotic was able to enter the cells. Thus, most of the antibiotic remained in the extracellular fluid.

**Figure 2:**
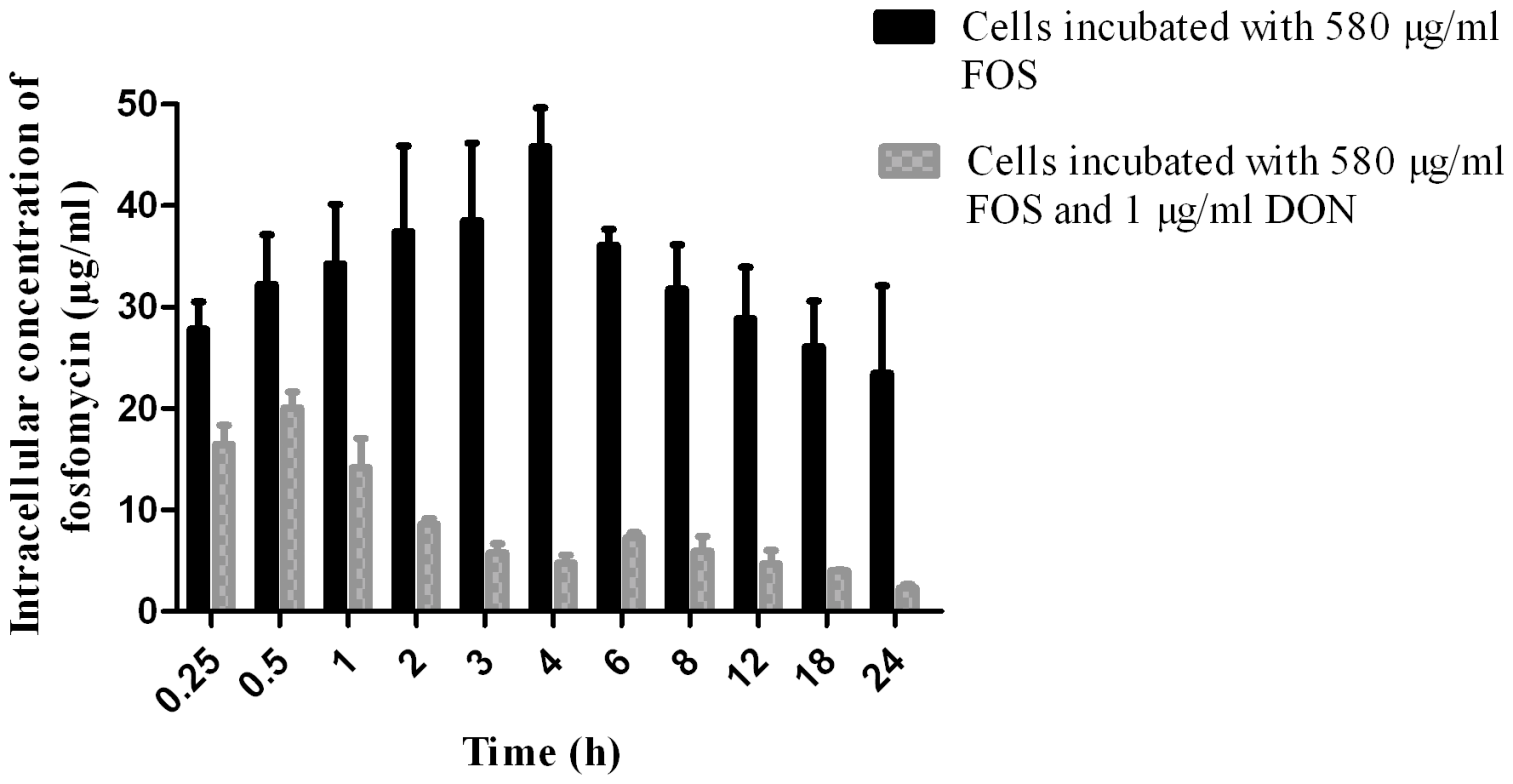
Intracellular concentration of FOS in IPEC-J2 cells exposed to FOS or the combination of FOS and DON during 24 h. Data are means of experiments performed in triplicate ± SD.

In the culture plates incubated with 580 µg/ml calcium FOS and 1 µg/ml DON, the C_max_ of intracellular FOS was 20.06 µg/ml vs. 45.81 µg/ml in the plates incubated only with the antibiotic (p<0.001). In the group incubated with both subtances, the C_max_ of intracellular FOS represented 3.46% in relation to the initial concentration of FOS. The concentration of intracellular antibiotic decreased up to 2.31 µg/ml at 24 h of incubation. Another important difference between both groups was the t_max_. In the presence of mycotoxin, the t_max_ was 30 min while in its absence, the t_max_ was 4 h. In summary, the C_max_ and t_max_ of FOS were lower in cells treated with FOS plus DON. As shown in figure 2, a significant effect of DON in the intracellular concentration of FOS was observed.

## Discussion

An important function of the gastrointestinal epithelium is to provide a barrier against the penetration of food contaminants and pathogens present in the intestinal lumen [30]. The disruption of the intestinal barrier allows an increased penetration of normally excluded luminal substances that may promote intestinal disorders [31], [32], [33]. In addition, the alteration of the intestinal epithelium interferes with the absorption of antibiotic. In the present study, we demonstrated that the intracellular concentration of FOS in IPEC-J2 cells decreased in the presence of low concentrations of DON.

Clinical effects associated with DON ingestion in pigs include feed refusal, weight loss, poor performance, gastrointestinal irritation (such as diarrhea, colic and rectal prolapse), squamous hyperplasia of the gastric lining and possible immunomodulation. The DON dose affecting feed intake and rate of gain in swine studies varied with the age of the pig, source of DON (purified versus natural contamination) and exposure conditions (*ad libitum* vs restrictive feeding and duration of exposure) [34]. Commonly, effects of DON have not been found at levels from 0.6 to 1 mg DON/kg feed [35]. For this experiment, the concentration of 1 ppm DON was established because it does not produce toxic effects *in vivo*. Pollmann et al. observed in 8 kg-pigs that when DON concentration was greater than 1 mg DON/kg in the diet, the feed intake declined [36]. In addition, Bergsjo et al. fed 25 kg-pigs a diet containing 2 and 4 mg DON/kg feed. The authors determined a non-effect level based on feed intake, weight gain and feed efficiency [37]. Furthermore, other reason to use 1 µg/ml of DON was based on the Commission Recommendation 2006/576/EC, which set down 0.9 mg/g of DON in complementary and complete feeding stuff for pigs as guidance value [38]. Moreover, no toxic effect was observed *in vitro* when IPEC-J2 cells were incubated with 1 µg/ml DON as determined by the trypan blue exclusion assay in which cell death was not observed after 24 h of incubation with the mycotoxin. These results are consistent with those observed by Vandenbroucke et al., who found that 1 µg/ml of DON was non-cytotoxic to differentiated IPEC-J2 cells [39]. However, Danicke et al. and Diesing et al. demonstrated that high concentrations of DON reduced the viability of porcine intestinal epithelial cells, especially when applied basolaterally [11], [24], [25].

FOS is a water soluble antibiotic, which is orally administered despite its poor bioavailability (F: 20±1.85% in pigs) [40]. The intestinal absorption of FOS occurs through both a saturable carrier-mediated mechanism and nonsaturable passive diffusion, as it was determined in rats [41], [42], [43], [44]. It is known that FOS has a hydrophilic structure, thereby; the passage of the drug by passive diffusion is not high [1], [45], [46], [47], [48]. An *in situ* intestinal perfusion study showed that carrier-mediated transport via a Na+-dependent phosphate (NaP_i_-II) is relevant, especially at concentrations of less than 1 mM FOS. The initial uptake of FOS was inhibited by phosphate in a concentration–dependent manner. On the other hand, an overshoot uptake of FOS was observed in the presence of an inwardly direct Na+ gradient [49], [50].

We have demonstrated in culture of HEp-2 cells that the intracellular concentration of FOS is not significantly modified when the cells are incubated with a concentration of 130 and 280 µg/ml FOS [51], [52]. Our data suggest that the concentration of 580 ppm FOS (> 1 mM) causes the saturation of this carrier and that 7.89% of the antibiotic was able to enter the cells.

The phosphate transporter carriers are mainly located in jejunum, being less active in ileum [49]. Therefore, FOS absorption would be limited to these intestinal segments. The saturable carrier-mediated mechanism of FOS transport across the enterocyte may impact on the pulse therapy administration of FOS in pig production. FOS is administered in pulse dosing in water twice/day. The residence time of intestinal digest in the FOS absorption site is only 3–4 h in pig [53]. Therefore, an important FOS pulse dose is lost through feces (F: 20±1.85%) [40].

The FOS oral dosage in water for pigs should consider the oral administration of small concentrations of the antibiotic along the day (according to physiological intake of water) to avoid the saturation of carrier phosphate. Beside, FOS is an antibiotic classified as “time dependent” and thereby adaptable to this method of administration [54].

It is known that DON affects epithelial protein synthesis and function [55], [56]. It has been demonstrated that DON affects the activities of intestinal transporters such as the sodium-dependent glucose/galactose transporter SGLT1, facilitated sugar transporters GLUT and amino-acid transports [57]. Maresca et al. reported that low concentrations (<10 µmol/L) of DON applied to human intestinal epithelial cells selectively modulate the activities of specific intestinal transporters, including the D-glucose and D-galactose sodium-dependent transporter, SGLT-1 and D-fructose transport GLUT5 [58]. Although it has not been demonstrated that DON modifies the sodium-ion-dependent phosphate transport which FOS uses to enter the cells, our findings suggest that the lower intracellular concentration of FOS in presence of the mycotoxin could be attributed to the effect of DON over these mechanisms.

In conclusion, our results indicate that a non-toxic concentration of DON on cells after short-term exposure interferes with the pharmacokinetic of FOS. Considering the frequent occurrence of DON in cereal-based foods and feeds worldwide, the importance of these findings should not be underestimated.
